# Strain Level *Streptococcus* Colonization Patterns during the First Year of Life

**DOI:** 10.3389/fmicb.2017.01661

**Published:** 2017-09-06

**Authors:** Meredith S. Wright, Jamison McCorrison, Andres M. Gomez, Erin Beck, Derek Harkins, Jyoti Shankar, Stephanie Mounaud, Edelwisa Segubre-Mercado, Aileen May R. Mojica, Brian Bacay, Susan A. Nzenze, Sheila Z. M. Kimaro, Peter Adrian, Keith P. Klugman, Marilla G. Lucero, Karen E. Nelson, Shabir Madhi, Granger G. Sutton, William C. Nierman, Liliana Losada

**Affiliations:** ^1^J. Craig Venter Institute Rockville, MD, United States; ^2^Research Institute of Tropical Medicine Muntinlupa City, Philippines; ^3^Respiratory and Meningeal Pathogens Research Unit Soweto, South Africa

**Keywords:** nasopharyngeal microbiome, *Streptococcus pneumoniae*, pneumococcal conjugate vaccine, Serotypes

## Abstract

Pneumococcal pneumonia has decreased significantly since the implementation of the pneumococcal conjugate vaccine (PCV), nevertheless, in many developing countries pneumonia mortality in infants remains high. We have undertaken a study of the nasopharyngeal (NP) microbiome during the first year of life in infants from The Philippines and South Africa. The study entailed the determination of the *Streptococcus* sp. carriage using a *lytA* qPCR assay, whole metagenomic sequencing, and *in silico* serotyping of *Streptococcus pneumoniae*, as well as 16S rRNA amplicon based community profiling. The *lytA* carriage in both populations increased with infant age and *lytA*+ samples ranged from 24 to 85% of the samples at each sampling time point. We next developed informatic tools for determining *Streptococcus* community composition and pneumococcal serotype from metagenomic sequences derived from a subset of longitudinal *lytA*-positive *Streptococcus* enrichment cultures from The Philippines (*n* = 26 infants, 50% vaccinated) and South African (*n* = 7 infants, 100% vaccinated). NP samples from infants were passaged in enrichment media, and metagenomic DNA was purified and sequenced. *In silico* capsular serotyping of these 51 metagenomic assemblies assigned known serotypes in 28 samples, and the co-occurrence of serotypes in 5 samples. Eighteen samples were not typeable using known serotypes but did encode for capsule biosynthetic cluster genes similar to non-encapsulated reference sequences. In addition, we performed metagenomic assembly and 16S rRNA amplicon profiling to understand co-colonization dynamics of *Streptococcus* sp. and other NP genera, revealing the presence of multiple *Streptococcus* species as well as potential respiratory pathogens in healthy infants. A range of virulence and drug resistant elements were identified as circulating in the NP microbiomes of these infants. This study revealed the frequent co-occurrence of multiple *S. pneumoniae* strains along with *Streptococcus* sp. and other potential pathogens such as *S. aureus* in the NP microbiome of these infants. In addition, the *in silico* serotype analysis proved powerful in determining the serotypes in *S. pneumoniae* carriage, and may lead to developing better targeted vaccines to prevent invasive pneumococcal disease (IPD) in these countries. These findings suggest that NP colonization by *S. pneumoniae* during the first years of life is a dynamic process involving multiple serotypes and species.

## Introduction

Invasive pneumococcal disease (IPD) caused by *Streptococcus pneumoniae* has decreased significantly after implementation of the pneumococcal conjugate vaccine (PCV) (Pilishvili et al., 2010; Tocheva et al., 2011). However, nasopharyngeal carriage of the pneumococcus in children <5 years old appears to continue at roughly 20–30% of the population in the US or Europe (Weatherholtz et al., 2010; Sharma et al., 2013; Fleming-Dutra et al., 2014; Lee et al., 2014). Carriage in low and middle income countries is higher with a pooled average of ~65% (Adegbola et al., 2014) and up to 75% in South Africa (Nzenze et al., 2014). Results from epidemiologic surveys show that the incidence of capsular serotypes targeted by the vaccine (VT) has decreased, while non-VT serotypes have increased (Huang et al., 2005; Pelton et al., 2007; Sharma et al., 2013). In particular, evidence is emerging that the serotypes targeted in the current vaccines include a lower fraction of the serotypes causing IPD in young children particularly in Asia and Africa compared to the protection afforded young children by the vaccines in developed countries (Hausdorff et al., 2000).

Detection of *S. pneumoniae* in clinical samples has traditionally been performed using microbiological cultures (Reller et al., 2008) or more recently, by quantitative PCR targeting the autolysin (*lytA)* gene (Messmer et al., 2004; WHO and CDC, 2011). In addition to detection of the organism from clinical samples, it is important to characterize the capsular serotype, since it has been shown that VT isolates are more likely to cause invasive disease than non-VT isolates (Weatherholtz et al., 2010; Fleming-Dutra et al., 2014). Capsule type is determined by serology using standardized antisera (Reller et al., 2008) or by multiplex PCR approaches that are able to discriminate between 20 and 37 of the more than 90 known capsule types (Satzke et al., 2013). However, these methods are laborious and expensive, and they have the inherent shortcoming that they cannot easily detect several capsular types in a single sample (Satzke et al., 2013). Methods that use high-throughput DNA sequencing have been presented as alternatives for capsular typing (Leung et al., 2012; Ip et al., 2014). These methods have relied on using a PCR enrichment step where the capsule loci are preferentially amplified directly from clinical samples, and thus suffer from similar limitations as multiplex PCR strategies. A more recent typing scheme using reads from whole genome sequence (WGS) data was developed to assign an *in silico* serotype (Kapatai et al., 2016). Here, we expand on the WGS approach using whole-metagenome sequencing of *Streptococcus*-enriched cultures and simultaneous development of bioinformatics approaches that clearly identify the capsular type. Our study demonstrates that metagenomics methods for serotyping *S. pneumoniae* directly from infant samples provide the potential for determining capsule information, the presence of other NP colonizers, and for providing data relating to virulence and drug resistance carriage.

## Materials and methods

### Study design and subjects

This study was performed in healthy infants whose mothers delivered at the Research Institute of Tropical medicine associated clinic in Muntinlupa City, Philippines or Chris Hani Baragwanath Hospital in Johannesburg, South Africa between June 2012 and January 2013. All mothers attending the clinics during the recruitment periods at each location were invited to participate in the study and written consent was obtained from all who agreed to participate. The study was approved by the Ethics Committees at both clinical sites and at the J. Craig Venter Institute (JCVI). Children were recruited to participate for 12 months. All of the children in South Africa were vaccinated against pneumococcus using PCV-7 according to the national vaccination schedule (Madhi et al., 2012). The Philippines had not implemented a national vaccination program against pneumococcus so half the children were randomly assigned to receive the PCV-10 vaccine (Rodenburg et al., 2010) vaccine.

### Nasopharyngeal sample collection and enrichment protocol

Sampling was performed according to each infant's scheduled visits: at birth (within 6 h), at the time of their first PCV vaccination (usually 6 weeks old), at the time of their second dose (usually at 14 weeks old), at the time of the last dose (40 weeks old), and at 12 months. Maternal samples were obtained at birth (only South Africa) and at 12 months (both sites). NP samples from infants and mothers were collected by pediatricians in the clinics using Copan Eswabs following manufacturer's instructions. After collection, samples were placed in 1 ml liquid Aimes buffer and stored on ice until delivery to the clinical laboratory. A 200 μl aliquot of NP sample was transferred to 6 ml Supplemented Todd-Hewitt Broth (THB) containing 0.5% yeast extract and 17% rabbit (Philippines) or fetal bovine (South Africa) serum and 10 mg/ml colistin and incubated at 37 °C at 5% CO_2_ without shaking for 6 h. Cells were then centrifuged at 9,000 rpm for 10 min and frozen at −20 C. Metagenomic DNA was extracted from this pellet using Qiagen DNeasy Blood and Tissue kit (Qiagen) following manufacturer's instructions. Purified DNA was transferred to QIAsafe DNA tubes (Qiagen), allowed to dry uncovered for 10–12 h in a laminar flow hood, and shipped to JCVI at ambient temperature.

### Definition of carriage by *lytA* Pcr

The presence of *S. pneumoniae* was assessed using a *lytA* qPCR as described (WHO and CDC, 2011) using primers F373: 5′-ACGCAATCTAGCAGATGAAGCA-3′ and R424: 5′ TCGTGCGTTTTAATTCCAGCT-3′. DNA was amplified using the following program: 95°C for 10 min, followed by 95°C for 15 s, 60°C for 1 min using TaqMan Universal Master Mix on a Biorad CFX96 Real-Rime PCR machine (RITM) or Applied Biosystems 7500 Real-Time PCR system(RMPRU). Samples were considered *lytA*-positive if the C_t_ value was below 35 (WHO and CDC, 2011).

### Metagenomic DNA sequencing

Only a subset of *lytA*-positive samples was selected for metagenomic sequencing, where infants were sampled at random with the goals to obtain *lytA-*positive samples for each representative age and following the pneumococcal population in a subset of infants for the duration of the study. Genomic DNA sequencing libraries were generated using standard library construction (Illumina), adding sample specific barcodes. Sequencing was performed by pooling 8–22 samples in a single 2 × 250 or 2 × 300 MiSeq run to obtain ~35 million reads per run.

### Metagenomic assembly pipeline

A pipeline to assemble reads and evaluate assembly content was developed as follows: (1) reads were adaptor and quality trimmed using trimmomatic (Bolger et al., 2014); (2) reads that mapped to the human reference genome GRCh38 (GCA_000001405.15) using bowtie2 version 2.2.7 (Langmead and Salzberg, 2012) with “sensitive” settings were removed; (3) filtered reads were then assembled with metaSPAdes version 3.7.1 (arXiv:1604.03071); and (4) BLAST-based evaluation of taxonomic and serotype content (details below) was conducted across metaSPAdes assembled contigs.

### Assembly-based and read-based taxonomic analysis

In order of execution, contigs larger than 200 bp from each metagenomic assembly were aligned against (1) a database of common Streptococcus genomes to identify intended host targets (alignments greater than 95% identity); and (2) the human reference GRCh38 to remove ancillary human contigs (alignments greater than 90% identity). Finally, the remaining set of contigs were aligned to the NCBI NT Bacterial Database (ref, link) BLASTN matches with >97% identity over 5% of the contig length were considered a match. The filtered BLASTN output from each sample were combined and then queried to identify the predominant taxa present in the enrichments by compiling all of the occurrences of a given reference genome across the samples. This genome list was then used to build a reference nucleotide database for read-mapping to more quantitatively assess the relative abundance of each taxa in the enrichment samples (Table S1). The database also included all finished *S. pneumoniae* genomes. Metagenomic reads were mapped using bowtie2 with very-sensitive settings such that reads could only map once to the reference taxonomic database. Counts of mapped reads to each genome were quantified and were used to assess the relative abundance in different samples.

### 16S rRNA community analysis of the non-enriched NP microbiome

To determine the pre-enrichment NP bacterial community composition, 16S rRNA amplicon profiling was performed on the initial sample before the enrichment step. Operational taxonomic units (OTUs) were generated *de novo* from raw Illumina sequence reads using an *in-house* analyses pipleline relying on the UPARSE (Edgar, 2013) and mothur (Schloss et al., 2009) open-source bioinformatics tools. Briefly, paired-end reads were trimmed of adapter sequences, barcodes, and primers prior to assembly, followed by discarding low quality reads and singletons. After a de-replication step and abundance determination, sequences were filtered for chimeras and clustered into OTUs. To assign taxonomy, we used the Wang classifier, and bootstrapped using 100 iterations. We set mothur to report full taxonomies only for sequences where 80 or more of the 100 iterations were the identical (cutoff = 80). Taxonomies were then assigned to the OTUs with mothur using version SSU Ref NR 99 version of the SILVA 16S ribosomal RNA database (Quast et al., 2013) as the reference. Tables with OTUs and the corresponding taxonomy assignments were generated and used in subsequent analyses. The resulting matrices were summarized by frequency across species-level resolution.

### Assembly-based *in silico* capsular and multi-locus sequence typing

The first step for establishing *in silico* method for serotyping was to create a nucleotide database of serotype sequences. Serotypes were assumed to be predominantly driven by the capsule polysaccharide (*cps*) locus of the *Streptococcus* strains. Capsule sequence exemplars were retrieved for all known serotypes from Bentley et al. (2006) and Skov Sorensen et al. (2016). Assemblies were aligned to this reference serotype nucleotide database for *in silico* serotyping using BLASTn. Sequence alignments greater than 98% identity over 2,000 bp were kept, and top matches of the cumulative alignment length for each serotype were identified via manual curation because in some cases multiple top matches were identified when more than one serotype was present. This was evident by cases in which different contigs had top matches to different serotypes. If no match was identified, metagenomic assemblies were then queried with *aliA* (NP_357921.1) and *dexB* (NP_357904.1), the two conserved genes upstream and downstream of *cps* cluster. The sequence region between these two flanking genes was then extracted from each metagenome assembly and evaluated by BLAST against the nucleotide non-redundant nt/nr database at NCBI to identify the match with the top total score. Multi-locus sequence typing (MLST) was performed on each metagenomic assembly *in silico* using LOCUST (Brinkac et al., 2017) using the *S. pneumoniae* MLST scheme at https://pubmlst.org/spneumoniae (Jolley and Maiden, 2010).

### Virulence and antibiotic resistance gene analysis

Contigs from metagenomic enrichment analysis were compared using BLAST alignments against a reference databases containing known antibiotic resistance determinants or virulence factors including *S. pneumoniae*-specific virulence genes (Zhou et al., 2007; Kadioglu et al., 2008; Liu and Pop, 2009; Mitchell and Mitchell, 2010; Chen et al., 2012; Blumental et al., 2015). BLAST results were filtered for hits that were greater than 90% identical over 80% of the reference length.

## Results

### *lytA*-positive burden in South Africa and Philippine infants

A total of 393 nasopharyngeal (NP) samples from 203 infants enrolled in our pediatric microbiome study were analyzed for *lytA* carriage as a proxy for *S. pneumoniae* colonization (Table 1). Most samples represented the first sample immediately after birth, the 6-, and 14-, 40-week, and 12 months since these corresponded to the pediatric visits when the PCV vaccine was administered or were the end-point of the microbiome project. After culture enrichment, the proportion of *lytA*-positive samples (C_T_ < 35) increased consistently with infant age, ranging from as low as 23.7% at birth to consistently above 85% after 7 months, with very little difference in the *lytA*-positive rates between the Philippines and South Africa, irrespective of vaccination status. Mother carriage of *lytA*-positive samples in South Africa was ~45% while *lytA* carriage from mothers in the Philippines was nearly 100%.

**Table 1:** Summary of *lytA* tested samples.

**Infant age**	**Site**	**Negative**	**Positive**	**Percent Positive**
Birth	Philippines	2	1	33.3
	South Africa	44	14	23.7
6 weeks^*^	Philippines	32	13	28.9
	South Africa	22	19	46.3
3 months^*^	South Africa	6	14	70
4 months^**^	Philippines	6		79.3
6 months	South Africa	1	4	80
7 months	South Africa	7	22	95.6
8 months	South Africa	2	12	86
9 months	South Africa		2	100
10 months^*^	Philippines	10	59	85.5
	South Africa	1	10	91
11 months	South Africa	1	8	89
12 months	Philippines	1	30	95.6
	South Africa		14	100
13 months	South Africa		10	100
15 months	South Africa	1	1	50
Total		137	256	61.5

* *PCV administration in Philippines and South Africa*.

** *PCV administration in Philippines*.

*Positive samples were defined as those with qPCR Ct values < 35 cycles*.

We obtained longitudinal time points for 93 subjects ranging from 2 to 7 samples per infant (average 3 samples). Thirty-three (35%) of those infants had *lytA-*positive samples every time they were sampled, including their earliest visit (Table 1). Of the remaining infants with longitudinal samples, 54 had negative *lytA* samples in their early visits and became *lytA-*positive over time, following the overall trend described above. The remaining six infants had negative *lytA* samples each time they were tested, though all but 2 of these samples corresponded to less than 2 months of age, again suggesting that the carriage and abundance of *lytA*-positive organisms is low at a very young age.

### Metagenomic sequencing and analysis of streptococcal carriage

A total of 51 samples were selected for further characterization through metagenomic sequencing in order to identify the various strains colonizing the NP of infants in each country. Samples were selected to represent primarily infants who had the maximum number of longitudinal *lytA*-positive samples in order to determine the effect of vaccination on pneumococcal population dynamics. Twelve samples were obtained from seven South African infants and 39 samples from 25 Philippine infants. Roughly one-half of the samples belonged to longitudinal samplings (Table 2). The majority of samples encoded multiple *lytA* genes in the metagenomic assembly of at least 80% nucleotide identity to the *S. pneumoniae* reference *lytA* sequence (NP_359346.1) (range: 1–4 copies, Table 2).

**Table 2:** Metagenomic enrichment sample characteristics.

							***# of lytA*** **(NP_359346.1) copies**			***In silico*** **serotyping (approximate coverage)**		
**Sample**	**Infant**	**Age (months)**	**Total Reads**	**# of *S. pneumoniae* Contigs**	***S. pneumoniae* Assembled Length**	**MLST ST**	**>95%**	**>90%**	**>80%**	**Serotype 1**	**Serotype 2**	**# non-encapsulated sequences**
RITM002I4	RITM002	4	2,196,659	606	2324313		1	2		23f (34x)	15a (19x)	0
RITM003I4	RITM003	4	1,449,077	1424	4108301			3		23a		0
RITM004I4	RITM004	4	1,160,995	724	4031720				3	45		2
RITM008I4	RITM008	4	1,190,829	737	3305375				2	nt		2
RITM009I10	RITM009	10	642,274	653	3255034			1	2	14		1
RITM019I10	RITM019	10	603,977	243	2169452		1		1	18c		0
RITM020I4	RITM020	4	2,267,733	468	3708044	771	1	1		6a		1
RITM020I10	RITM020	10	1,591,241	1444	4769031		1	1	2	nt		2
RITM020I12	RITM020	12	1,217,954	835	3059123				2	nt		1
RITM021I10	RITM021	10	6,470,947	830	3869427	383	1		2	16f		1
RITM022I4	RITM022	4	1,113,057	718	4101518		1	1	2	15f		1
RITM022I12	RITM022	12	1,358,394	724	3245844			1	1	nt		1
RITM023I4	RITM023	4	1,533,815	525	4227905		1	1	2	6b		0
RITM029I10	RITM029	10	2,479,318	1000	4431671		1		2	5b (151x)	15b/c (50x)	1
RITM034I10	RITM034	10	1,326,646	614	2491075		1		1	nt		1
RITM034I12	RITM034	12	1,508,905	432	4187807		1		1	19f		1
RITM038I10	RITM038	10	739,843	515	3831006		1	1	2	19f		1
RITM042I10	RITM042	10	1,380,897	805	2914729		1		2	nt		1
RITM043I2	RITM043	6 weeks	3,162,069	1228	3460826		1	1		~6b		0
RITM046I2	RITM046	6 weeks	2,477,879	423	2836698			1		nt		1
RITM052I2	RITM052	6 weeks	3,110,195	641	2642259		1		2	nt		2
RITM052I4	RITM052	4	1,854,279	266	2252249	42	1			23a		1
RITM052I12	RITM052	12	452,425	641	2642259		1		2	23a (40x)	45 (40x)	0
RITM053I10	RITM053	10	803,332	1498	3115520			1	1	nt		1
RITM053I12	RITM053	12	1,152,462	704	4467827		1	1	2	6a		1
RITM059I10	RITM059	10	642,400	565	3376556	473	1			6b		1
RITM059I12	RITM059	12	566,500	481	3142311	473	1			6b		1
RITM060I10	RITM060	10	859,223	514	2462829	Novel	1		1	23f		0
RITM060I12	RITM060	12	474,933	1242	3707253		1		2	45		0
RITM070I10	RITM070	10	347,804	871	2742804			1		nt		1
RITM070I12	RITM070	12	521,883	730	3308305		1	1		19a		1
RITM071I10	RITM071	10	1,400,393	829	4529939		1		2	19f		0
RITM071I12	RITM071	12	1,954,642	652	5186483		1			11f (109x)	19c (22x)	0
RITM077I2	RITM077	6 weeks	1,670,013	553	3159554	4745	1			20 (103x)	19c (11x)	1
RITM077I10	RITM077	10	2,614,423	348	3178458		1	1	1	6a		0
RITM081I10	RITM081	10	1,293,683	829	4529939		1	1		nt		2
RITM081I12	RITM081	12	1,205,277	578	4613175		1	1	1	nt		2
RITM084I2	RITM084	6 weeks	1,523,208	729	2888646				1	nt		2
RITM089I4	RITM089	4	373,100	617	2723492		1		1	10a		0
RMPRU004I4	RMPRU004	4	1,813,223	1673	2044289		1			nt		0
RMPRU004I7	RMPRU004	10	934,919	81	2110604	9811	1	1		nt		1
RMPRU008I4	RMPRU008	4	1,645,268	1202	3059009				3	nt		0
RMPRU008I7	RMPRU008	10	885,194	768	2456582			1	1	nt		1
RMPRU010I3	RMPRU010	6 weeks	8,554,212	102	2088121	10550	1		1	15a		0
RMPRU010I7	RMPRU010	10	1,733,329	1447	3206902		1	1	1	16f		0
RMPRU011I4	RMPRU011	6 weeks	1,688,331	125	2100736	4088	1	1		16f		1
RMPRU011I7	RMPRU011	10	2,262,503	520	2329245				1	nt		0
RMPRU011I9	RMPRU011	10	1,273,109	742	4428067		1	1	1	16f		1
RMPRU022I12	RMPRU022	12	467,655	1180	639982				2	19c		1
RMPRU023I2	RMPRU023	6 weeks	677,044	460	2680898	8334	1			23f		0
RMPRU031I2	RMPRU031	6 weeks	710,789	222	2147330	5647	1	1		13		0

*RITM, the Philippines; RMPRU, South Africa. S. pneumoniae contigs and assembled length determined by BLAST match to S. pneumoniae reference genomes for each contig (see text for details). Multilocus sequence typing (MLST) sequence types (ST) were assigned using the PubMLST database. The number of lytA copies in each assembly is based on % identity thresholds relative to the S. pneumoniae R6 copy (NP_359346.1). Serotype assignment was generated from in silico typing of WGS metagenomic assemblies (see text for details). nt, non-typeable*.

### Population structure of nasopharyngeal *Streptococcus* community

**Figure 1:**
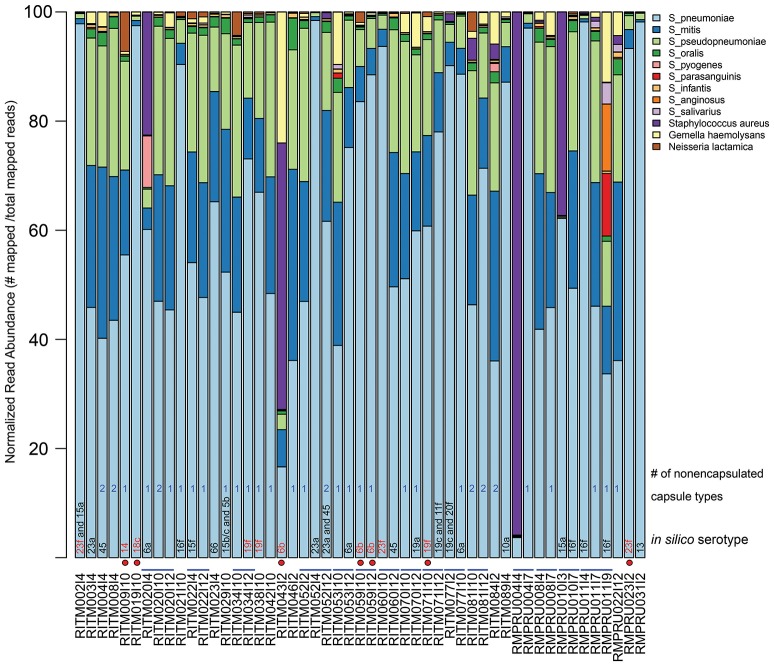
Relative abundance of NP microbiome taxa from metagenomic analysis of enrichment cultures. Abundance is based on normalized read counts mapped to a reference database of *Streptoccocus* species and other taxa detected in the NP enrichment assemblies (Table S1). The sample names that indicate infant and sampling time point are provided under the x-axis. Blue lines connecting sample names highlight longitudinal samples originating from the same infant. *In silico* serotype classification was assigned using a BLAST-based strategy by aligning metagenomic assemblies against a reference database of capsule biosynthetic loci (see Methods for details). Red-colored serotype text indicates a vaccine-type serotype while a red circle depicts which vaccine-type serotype samples came from vaccinated infants. A count of the number of contigs aligning to the nonecapsulated NT_110_58-like capsule locus is given (see text for details).

Our metagenomic approach to studying *Streptococcus* spp. colonizing the nasopharynx allowed a very detailed view of the various organisms that reside in that space. The taxonomic composition of the enriched NP microbiome based on percentage of mapped reads to various reference genomes indicated the predominance of *S. pneumoniae* in most samples (Figure 1, Table S2, mean: 61.6%, range: 3.7–98.4%). Other common *Streptococcus* taxa include S. mitis (mean: 14.9%), *S. pseudopneumoniae* (mean: 14.4%), *S. oralis* (mean: 1.2%). Other *Streptococcus* sp. were detected at >5% in a limited number of infants: *S. pyogenes* (1 infant: 9.4%), *S. parasanguinis* (1 infant: 11.5%), *S. anginosus* (1 infant, 12.2%). One NP sample (RMPRU011I9) had the most diverse *Streptococcus* community comprised of four species with >10% mapped reads, though the previous two samples from that infant were comprised of primarily *S. pneumoniae* and *S. mitis* and *S. pseudopneumoniae*. Other taxa present in the enrichments include *Staphylococcus aureus* (4 samples >5% reads, range: 0–96%), *Gemella haemolytica* (5 samples >5%, range: 0–12.8%), and *Neisseria lactamica* (1 sample >5%, range: 0–7.2%). The diverse *Streptococcus* sample (RMPRU011I9) also had a substantial number of *Gemella* reads in stark contrast to the previous two samples from the infant.

**Figure 2:**
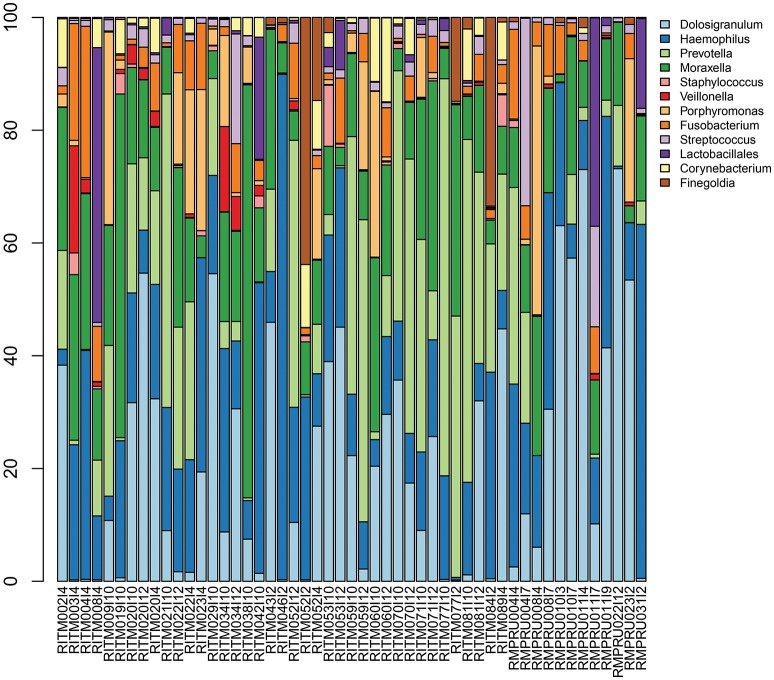
Infant nasopharyngeal microbiome taxonomic composition based on 16S rRNA amplicon sequencing of the pre-enrichment sample. Taxa with >5% relative abundance in at least one sample are depicted.

*Streptococcus* sp. 16S rRNA amplicon sequences comprised between <1 and 33% of reads (Figure 2, Table S3) from the initial NP microbiome sample. The community composition varied greatly in relative abundance of different taxa, but the primary taxa was largely consistent with *Dolosigranulum, Haemophilus, Prevotella, and Moraxella* being the most prevalent. Other taxa prevalent in a fewer number of samples include *Porphyromonas, Finegoldia, and Johnsonella*.

### Capsular type detection and serotype prediction

We applied *in silico* BLAST-based methods to ascertain the capsular type(s) present in these metagenomic samples. Using a criteria of >98% nucleotide identity, 33 samples were assigned a serotype. The most common serotype was 16f (four samples) while the following were encountered three times: 6a, 6b, 16f, 19c, 19f, and 23a (Figure 1, Table 2). The presence of more than one serotype was detected in five infants. Ten samples from The Philippines cohort had capsule types belonging to PCV10 vaccine types, and five of those samples originate from vaccinated infants (i.e., RITM009, RITM059, and RITM071), most of which occurred in infants >10 months of age. However, one VT-serotype sample originated from a 6-week-old infant (RITM043I2). One vaccinated South African infant carried a vaccine type serotype (23f) at 6 weeks, the timepoint for the first PCV7 administration. For longitudinal samples originating from the same infant, only three infants had the same capsule type at more than one visit (RITM052:23A, RITM059:6b, and RMPRU011:16f).

The samples without *in silico* serotype matches were further interrogated to determine whether nontypeable *Streptococcus* capsule biosynthetic genes were present by examining sequence content between *aliA* and *dexB*, the conserved genes flanking the capsule biosynthetic cluster. The majority of extracted capsule sequences matched at 94–96% identity to several variants of the capsule locus detailed in Park et al. (2012) including the complete *S. pneumoniae* NT-110-58 genome (CP007593) (Hilty et al., 2014) (Table 2), as well as the complete genomes of *S. mitis* B6 (FN568063.1) and *S. pseudopneumoniae* IS7493 (CP002925.1) (Shahinas et al., 2011). Similar sequences (>94% similarity) were also present in the serotypeable samples as well (Figure 1) indicating that they are prevalent and co-exist with *S. pneumoniae* serotypes.

### *In silico* MLST analysis

MLST types were definitively assigned from the metagenomes of twelve samples (Table 2, Table S4), two of which were from the same infant (RITM059) with the same MLST type (ST473). One additional samples (RITM060I10) represented a novel sequence type comprised of previously classified alleles. South African sequence types matched other ST from South African in the PubMLST isolate database, while Philippine samples were comprised of sequence types from more diverse locations.

### Virulence factors genes

We predicted metagenomes from *S. pneumoniae* samples to encode core virulence factors *lytA, ply* (pneumolysin), *nanA* (neuraminidase A), *hyl* (hyaluronidase), *pspC* (pneumococcal surface protein C), and *pavA* (pneumococcal adhesion and virulence A) (Hiller et al., 2007). All samples encoded at least one *S. pneumoniae* virulence factor when compared to reference databases (Table S5; Zhou et al., 2007; Chen et al., 2012). The five representative virulence factors examined here were present in >50% of the metagenomes, where *ply* was present in almost all samples (98%). Several samples encoded more than one sequence distinguishable copy of *hyl, ply*, and *pavA*. One sample that contained both *S. aureus*, and *S. pyogenes* encoded a total of 62 virulence factors, including both staphylococcal and streptococcal toxins and complement evasion factors (Tables S5, S6). The majority of *Staphylococcus-*containing samples had more than 10 virulence factors including haemolysins and toxins, indicating the presence of fully virulent *S. aureus* (Powers and Wardenburg, 2014).

### Antibiotic resistance markers

Twelve samples contained antibiotic resistance genetic determinants (Table S7): nine samples from the Philippines and three from South Africa. Seven samples encoded only one antibiotic resistance marker and two samples encoded 6 or more. Metagenome assemblies from two samples encoded the *bla*(TEM-1) gene, which is the most common β-lactamase in Gram-negative bacteria (Muhammad et al., 2014). The gene was encoded in contigs with relatively low read coverage was highly similar to *Neisseria* plasmids (Muhammad et al., 2014). Both TEM-1 samples were obtained from Philippine infants, one from a 6-week visit (RITM077), and the other from the 12-month visit (RITM022). One sample from a 6-week-old infant (RMPRU023I2) encoded the methicillin-resistance gene, *mecA*. The *mecA* gene was surrounded by sequences homologous to the transponson involved in *mecA* mobilization (Katayama et al., 2001), suggesting it was encoded by a mobile element.

## Discussion

In this study, we report the use of targeted culture enrichment and metagenomic sequencing to study the dynamics of *Streptococcus* carriage in the infant nasopharynx in the Philippines and South Africa. A total of 393 samples from 203 infants were analyzed, where the majority of early samples were *lytA*-negative which is consistent with other studies and with colonization occurring later in life (>4 months) (Coles et al., 2001; Ercibengoa et al., 2012; Turner et al., 2012). Broth enrichment culture has been demonstrated to be a powerful approach to increasing the sensitivity for detecting the carriage of *S. pneumoniae* in the upper respiratory tract. When methods are compared on the same samples, the carrier fraction of the samples and the serotype diversity are maximal for the broth enrichment culture (da Gloria Carvalho et al., 2010). Metagenomic sequencing of the entire enrichment culture allowed us to see the range of bacteria that were selected by the enrichment culture protocol. The assembly data suggested that streptococcal enrichment was successful, with *Streptococcus* sp. reads accounting for an average of 2% of the 16S rRNA reads from the pre-enriched NP community, to an average of 93% of post-enrichment mapped reads. All samples had more than one *Streptococcus* sp. present including *S. pseudopneumoniae* and *S. mitis*. The detection of multiple *lytA* sequences of varying nucleotide similarity supports the idea that the NP community is colonized by a complex assemblage of *Streptococcus* organisms. This observation highlights the potential for genetic exchange among closely related *Streptococcus* sp. as recombination is a well-characterized mechanism for generating genetic diversity within the species (Hanage et al., 2009; Chaguza et al., 2015, 2016). Among the other taxa identified genera by 16S rRNA gene analysis in the non-enriched primary sample, were common NP microbiome taxa including *Dolosigranulum, Haemophilus, Moraxella*, and *Prevotella* sequences (Bogaert et al., 2011; Perez-Losada et al., 2017). Some studies have suggested that *Corynebacterium* and *Dolosigranulum* presence are protective from *S. pneumoniae* colonization (de Steenhuijsen Piters and Bogaert, 2016), but the limited sample size and general low prevalence of *S. pneumoniae* in this 16S rRNA data precludes much inference about the relationship. Other taxa enriched in the metagenomic analysis include *Staphylococcus, Gemella*, and *Neisseria* indicating that the enrichment protocol shifted the community composition substantially.

The use of *lytA* for detecting pneumococcus in community acquired pneumonia cases has been documented and is frequently employed as a rapid assay (Abdeldaim et al., 2010). In this study where the subjects were largely free of respiratory infections, the *lytA* assay detected the presence of *S. pneumoniae* as a member of the commensal microbiome but also detected other *lytA* containing streptococcal species in the commensal NP microbiome. Recent screening assays have in fact documented that *lytA* is not a specific diagnostic gene for *S. pneumoniae* (Simoes et al., 2016). Undoubtedly the use of a second pneumococcus selective gene would greatly improve the specificity of the assay for use as a rapid pneumococcus diagnostic tool for respiratory infections.

Although the presence of *Streptococcus* spp. in the nasopharynx of these infant subjects was both common and frequent, it was relatively uncommon for a child to have consistent colonization by the same *S. pneumoniae* strain. There were only three instances of the same capsular type in samples obtained over 3 months apart. Studies of serotype switching have been focused on such switching events in the context of PCV vaccination (for example see Hanage et al., 2011) but not in such young children. Serotypes related to the vaccine (PCV10 in the Philippines and PCV7 in South Africa) were observed in 11 samples, seven of which came from vaccinated infants. However, two of these samples originated from infants on their first scheduled vaccine administration, while the other five samples came from infants >10 months of age. This highlights the need for further examination of vaccine success in these populations. Multiple samples also had more than one serotype present concurrently, and many encoded both typeable and non-typeable capsule loci. This is consistent with previous studies using different methods, and again highlights the potential for genetic exchange between *Streptococcus* strains (Kamng'ona et al., 2015). *In silico* MLST typing indicates that many samples were not typeable, but for those that were, only one infant had the same sequence type more than once (Table 2). The remaining samples could not be specifically assigned to a single MLST type either because the assembly did not resolve all the loci necessary for typing especially in those cases with co-occurring *S. pneumoniae*, or because loci had no matches compared to known MLST types.

The 16S rRNA NP longitudinal sampling demonstrated consequential variation between successive samples for the NP community composition in our infants during their first year of life. It is likely that the serotype variation we are observing is a consequence of the inherent instability of the NP microbiome during this early stage of life (Jebaraj et al., 1999; Hohwy et al., 2001; Turner et al., 2011; Ercibengoa et al., 2012). Another striking observation on the NP microbiomes in these infants is the prevalence of potentially pathogenic species acting as commensal members of the young infant NP microbiome. We have noted the presence of pathogenic bacteria in the respiratory tract microbiome of lung transplant patients in the absence of an infection, and often when these patients did present with a pneumonia, the pathogen was earlier detectable as a prior member of the commensal population before the onset of disease (Shankar et al., 2015). In this context it is not surprising that we detected the presence of at least one *S. pneumoniae* virulence factor in all of the metagenomic enrichment culture samples, with the majority of *Staphylococcus*-containing samples exhibiting more than 10 virulence factors. Furthermore, our detection of antibiotic resistance genes and mobile elements that can be easily transferred between strains suggests that the infant NP serves as a reservoir for antibiotic resistant potential. These observations are consistent with a hypothesis that in these young infants, potentially pathogenic bacteria are common members of the commensal microbiome and that bacterial respiratory disease does not simply result from the presence of a bacterial respiratory pathogen but is the result of a more complex interaction between the host immune system status and the respiratory tract microbiome. However, the mechanisms behind the activation and phenotypic manifestation of virulence in the early NP microbiome remain unclear.

## Conclusions

The *in silico* serotype approach here may contribute to serotype analysis of strains isolated from infants that could lead to better data on residual serotypes that constitute the reservoir for future pneumococcal infections post-targeted vaccines to prevent IPD in infants in these countries. In addition, the study revealed the frequent presence of bacterial pathogens in the NP microbiome of these infants with genomes encoding an abundance of virulence and antibiotic resistance elements. Evidence is emerging that the serotypes targeted in the current vaccines are not as protective for young children in developing countries. The serotype tool reported here may contribute to serotype analysis of strains isolated for infants with IPD that could lead to developing better targeted vaccines to prevent IPD in infants in these countries.

## Ethics statement

The study was approved by the Ethics Committees at both clinical sites and at the J. Craig Venter Institute (JCVI). For the South African cohort, approval was issued by the University of Witwatersrand, Johannesburg Human Research Ethics committee on 2/24/12 and reviewed with approval on 8/6/2013. The J. Craig Venter Institute Institutional Review Board approval was issued on 2/4/2012. For the Philippine cohort, approval was issued on 2/28/2012 by the Research Institute for Tropical Medicine Institutional Review board, assigned number 2012-002. The J. Craig Venter Institute Institutional Review Board approval was issued on 4/4/2012.

## Availability of data

The WGS data supporting the conclusions of this article are available in GenBank under accession number PRJNA31170 http://www.ncbi.nlm.nih.gov/bioproject/PRJNA311705/. Other concluding datasets can be found within article and its additional files.

## Author contributions

LL and MW were the major contributors to study design, performed the analysis, and crafted the manuscript. JM, AG, EB, DH, and JS participated in software tool design and data analysis and performed the statistical analysis and interpretation of the data. StM managed the materials and data exchanges and interactions among the clinical site and JCVI and organized the metadata and participated in editing of the manuscript. ES, AM, BB, SN, SK, ML, and ShM Contributed to study design, and sample and data collection. SK and PA participated in laboratory testing. GS participated in software tool design, and data analysis as well as critically reading the manuscript. KK was instrumental in developing the collaborative interactions with the project's South Africa clinical site and contributed to the coordination of the project with the Philippine clinical site. He provided guidance to the serotyping study design and performed a critical review of the manuscript prior to submission. KN and WN participated in the study design, coordinated the project across the three collaborating sites, and participated in editing the manuscript.

### Conflict of interest statement

KK declares that he is currently employed by the Bill and Melinda Gates Foundation employee. The other authors declare that the research was conducted in the absence of any commercial or financial relationships that could be construed as a potential conflict of interest. The reviewer AG and handling Editor declared their shared affiliation.

## Supplementary material

Table S1 Accession information for reference *Streptococcus* species and other abundant taxa used to construct nucleotide database for metagenomic read mapping.

Table S2 Taxonomic composition of post-enrichment NP microbiome based on read mapping counts to reference database comprised of most abundant taxa in BLAST-based analysis of metagenomic assemblies.

Table S3 16S rRNA taxonomic composition of pre-enrichment NP microbiome.

Table S4 Multilocus sequence typing allelic profile for each sample assigned using the PubMLST database for *S. pneumoniae*.

Table S5 *S. pneumoniae* virulence factor genes.

Table S6 Virulence factor genes from other taxa.

Table S7 Antibiotic resistance genes.

## References

[B1] Abdeldaim G. Herrmann B. Mölling P. Holmberg H. Blomberg J. Olcén P.. (2010). Usefulness of real-time PCR for lytA, ply, and Spn9802 on plasma samples for the diagnosis of pneumococcal pneumonia. Clin. Microbiol. Infect. 16, 1135–1141. 10.1111/j.1469-0691.2009.03069.x 19832718

[B2] Adegbola R. A. DeAntonio R. Hill P. C. Roca A. Usuf E. Hoet B.. (2014). Carriage of *Streptococcus Pneumoniae* and other respiratory bacterial pathogens in low and lower-middle income countries: a systematic review and meta-analysis. PLoS ONE 9:e103293. 10.1371/journal.pone.0103293 25084351 PMC4118866

[B3] Bentley S. D. Aanensen D. M. Mavroidi A. Saunders D. Rabbinowitsch E. Collins M.. (2006). Genetic analysis of the capsular biosynthetic locus from all 90 pneumococcal serotypes. PLoS Genet. 2:e31. 10.1371/journal.pgen.0020031 16532061 PMC1391919

[B4] Blumental S. Granger-Farbos A. Moisi J. C. Soullie B. Leroy P. Njanpop-Lafourcade B. M.. (2015). Virulence factors of *Streptococcus Pneumoniae*. comparison between African and French invasive isolates and implication for future vaccines. PLoS ONE 10:e0133885. 10.1371/journal.pone.0133885 26214695 PMC4516325

[B5] Bogaert D. Keijser B. Huse S. Rossen J. Veenhoven R. van Gils E.. (2011). Variability and diversity of nasopharyngeal microbiota in children: a metagenomic analysis. PLoS ONE 6:e17035. 10.1371/journal.pone.0017035 21386965 PMC3046172

[B6] Bolger A. M. Lohse M. Usadel B. (2014). Trimmomatic: a flexible trimmer for illumina sequence data. Bioinformatics 30, 2114–2120. 10.1093/bioinformatics/btu170 24695404 PMC4103590

[B7] Brinkac L. M. Beck E. Inman J. Venepally P. Fouts D. E. Sutton G. (2017). LOCUST: a custom sequence locus typer for classifying microbial isolates. Bioinformatics 33, 1725–1726. 10.1093/bioinformatics/btx045 28130240 PMC5860141

[B8] Chaguza C. Andam C. P. Harris S. R. Cornick J. E. Yang M. Bricio-Moreno L.. (2016). Recombination in *Streptococcus pneumoniae* lineages increase with carriage duration and size of the polysaccharide capsule. MBio 7:e01053–16. 10.1128/mBio.01053-16 27677790 PMC5040112

[B9] Chaguza C. Cornick J. E. Everett D. B. (2015). Mechanisms and impact of genetic recombination in the evolution of *Streptococcus pneumoniae*. Comput. Struct. Biotechnol. J. 13, 241–247. 10.1016/j.csbj.2015.03.007 25904996 PMC4404416

[B10] Chen L. Xiong Z. Sun L. Yang J. Jin Q. (2012). VFDB 2012 update: toward the genetic diversity and molecular evolution of bacterial virulence factors. Nucleic Acids Res. 40, D641–D645. 10.1093/nar/gkr989 22067448 PMC3245122

[B11] Coles C. L. Kanungo R. Rahmathullah L. Thulasiraj R. D. Katz J. Santosham M.. (2001). Pneumococcal nasopharyngeal colonization in young South Indian infants. Pediatr. Infect. Dis. J. 20, 289–295. 10.1097/00006454-200103000-00014 11303832

[B12] da Gloria Carvalho M. Pimenta F. C. Jackson D. Roundtree A. Ahmad Y. Millar E. V.. (2010). Revisiting pneumococcal carriage by use of broth enrichment and PCR techniques for enhanced detection of carriage and serotypes. J. Clin. Microbiol. 48, 1611–1618. 10.1128/JCM.02243-09 20220175 PMC2863911

[B13] de Steenhuijsen Piters W. A. Bogaert D. (2016). Unraveling the molecular mechanisms underlying the nasopharyngeal bacterial community structure. MBio 7:e00009–16. 10.1128/mBio.00009-16 26838716 PMC4742699

[B14] Edgar R. C. (2013). UPARSE: highly accurate OTU sequences from microbial amplicon reads. Nat. Methods 10, 996–998. 10.1038/nmeth.2604 23955772

[B15] Ercibengoa M. Arostegi N. Marimon J. Alonso M. Perez-Trallero E. (2012). Dynamics of pneumococcal nasopharyngeal carriage in healthy children attending a day care center in northern Spain, influence of detection techniques on the results. BMC Infect. Dis 12:69. 10.1186/1471-2334-12-69 22440017 PMC3383471

[B16] Fleming-Dutra K. E. Conklin L. Loo J. D. Knoll M. D. Park D. E. Kirk J.. (2014). Systematic review of the effect of pneumococcal conjugate vaccine dosing schedules on vaccine-type nasopharyngeal carriage. Pediatr. Infect. Dis. J. 33, S152–S160. 10.1097/INF.0000000000000083 24336057 PMC3940522

[B17] Hanage W. P. Bishop C. J. Huang S. S. Stevenson A. E. Pelton S. I. Lipsitch M.. (2011). Carried pneumococci in Massachusetts children; the contribution of clonal expansion and serotype switching. Pediatr. Infect. Dis. J. 30, 302–308 10.1097/INF.0b013e318201a154 21085049 PMC3175614

[B18] Hanage W. P. Fraser C. Tang J. Connor T. R. Corander J. (2009). Hyper-recombination, diversity, and antibiotic resistance in pneumococcus. Science 324, 1454–1457. 10.1126/science.1171908 19520963

[B19] Hausdorff W. P. Bryant J. Paradiso P. R. Siber G. R. (2000). Which pneumococcal serogroups cause the most invasive disease: implications for conjugate vaccine formulation and use, part I. Clinical Infectious Diseases 30, 100–121. 10.1086/313608 10619740

[B20] Hiller N. L. Janto B. Hogg J. S. Boissy R. Yu S. Powell E.. (2007). Comparative genomic analyses of seventeen streptococcus pneumoniae strains: insights into the *Pneumococcal Supragenome*. J. Bacteriol. 189, 8186–8195. 10.1128/JB.00690-07 17675389 PMC2168654

[B21] Hilty M. Wuthrich D. Salter S. J. Engel H. Campbell S. Sa-Leao R.. (2014). Global phylogenomic analysis of nonencapsulated Streptococcus pneumoniae reveals a deep-branching classic lineage that is distinct from multiple sporadic lineages. Genome Biol. Evol. 6, 3281–3294. 10.1093/gbe/evu263 25480686 PMC4986459

[B22] Hohwy J. Reinholdt J. Kilian M. (2001). Population dynamics of *Streptococcus* mitis in its natural habitat. Infect. Immun. 69, 6055–6063. 10.1128/IAI.69.10.6055-6063.2001 11553543 PMC98734

[B23] Huang S. S. Platt R. Rifas-Shiman S. L. Pelton S. I. Goldmann D. Finkelstein J. A. (2005). Post-PCV7 changes in colonizing pneumococcal serotypes in 16 Massachusetts communities, 2001 and 2004. Pediatrics 116, e408–e413. 10.1542/peds.2004-2338 16140686

[B24] Ip M. Liyanapathirana V. Ang I. Fung K. S. C. Ng T. K. Zhou H.. (2014). Direct detection and prediction of all pneumococcal serogroups by target enrichment-based next-generation sequencing. J. Clin. Microbiol. 52, 4244–4252. 10.1128/JCM.02397-14 25274995 PMC4313294

[B25] Jebaraj R. Cherian T. Raghupathy P. Brahmadathan K. N. Lalitha M. K. Thomas K.. (1999). Nasopharyngeal colonization of infants in southern India with *Streptococcus Pneumoniae*. Epidemiol. Infect. 123, 383–388. 10.1017/S0950268899003131 10694148 PMC2810771

[B26] Jolley K. A. Maiden M. C. (2010). BIGSdb: scalable analysis of bacterial genome variation at the population level. BMC Bioinformatics 11:595. 10.1186/1471-2105-11-595 21143983 PMC3004885

[B27] Kadioglu A. Weiser J. N. Paton J. C. Andrew P. W. (2008). The role of *Streptococcus pneumoniae* virulence factors in host respiratory colonization and disease. Nat. Rev. Microbiol. 6, 288–301. 10.1038/nrmicro1871 18340341

[B28] Kamng'ona A. W. Hinds J. Bar-Zeev N. Gould K. A. Chaguza C. Msefula C.. (2015). High multiple carriage and emergence of *Streptococcus pneumoniae* vaccine serotype variants in Malawian children. BMC Infect. Dis. 15:234. 10.1186/s12879-015-0980-2 26088623 PMC4474563

[B29] Kapatai G. Sheppard C. L. Al-Shahib A. Litt D. J. Underwood A. P. Harrison T. G.. (2016). Whole genome sequencing of *Streptococcus pneumoniae*: development, evaluation and verification of targets for serogroup and serotype prediction using an automated pipeline. PeerJ 4:e2477. 10.7717/peerj.2477 27672516 PMC5028725

[B30] Katayama Y. Ito T. Hiramatsu K. (2001). Genetic organization of the chromosome region surrounding mecA in clinical staphylococcal strains: role of IS431-mediated mecI deletion in expression of resistance in mecA-carrying, low-level methicillin-resistant *Staphylococcus haemolyticus*. Antimicrob. Agents Chemother. 45, 1955–1963. 10.1128/AAC.45.7.1955-1963.2001 11408208 PMC90585

[B31] Langmead B. Salzberg S. L. (2012). Fast gapped-read alignment with Bowtie 2. Nat. Methods 9, 357–359. 10.1038/nmeth.1923 22388286 PMC3322381

[B32] Lee G. M. Kleinman K. Pelton S. I. Hanage W. Huang S. S. Lakoma M.. (2014). Impact of 13-valent pneumococcal conjugate vaccination on carriage in young children in massachusetts. J. Pediatric Infect. Dis. Soc. 3, 23–32. 10.1093/jpids/pit057 24567842 PMC3933044

[B33] Leung M. H. Bryson K. Freystatter K. Pichon B. Edwards G. Charalambous B. M.. (2012). Sequetyping: serotyping *Streptococcus pneumoniae* by a single PCR sequencing strategy. J. Clin. Microbiol. 50, 2419–2427. 10.1128/JCM.06384-11 22553238 PMC3405617

[B34] Liu B. Pop M. (2009). ARDB–antibiotic resistance genes database. Nucleic Acids Res 37, D443–D447. 10.1093/nar/gkn656 18832362 PMC2686595

[B35] Madhi S. A. Cohen C. von Gottberg A. (2012). Introduction of pneumococcal conjugate vaccine into the public immunization program in South Africa: translating research into policy. Vaccine 30, C21–C27. 10.1016/j.vaccine.2012.05.055 22939016

[B36] Messmer T. O. Sampson J. S. Stinson A. Wong B. Carlone G. M. Facklam R. R. (2004). Comparison of four polymerase chain reaction assays for specificity in the identification of *Streptococcus pneumoniae*. Diagn. Microbiol. Infect. Dis. 49, 249–254. 10.1016/j.diagmicrobio.2004.04.013 15313529

[B37] Mitchell A. M. Mitchell T. J. (2010). *Streptococcus pneumoniae*: virulence factors and variation. Clin. Microbiol. Infect. 16, 411–418. 10.1111/j.1469-0691.2010.03183.x 20132250

[B38] Muhammad I. Golparian D. Dillon J. A. Johansson A. Ohnishi M. Sethi S. (2014). Characterisation of bla TEM genes and types of beta-lactamase plasmids in *Neisseria gonorrhoeae*- the prevalent and conserved bla TEM-135 has not recently evolved and existed in the Toronto plasmid from the origin. BMC Infect. Dis. 14:454 10.1186/1471-2334-14-454 25149062 PMC4152594

[B39] Nzenze S. A. Shiri T. Nunes M. C. Klugman K. P. Kahn K. Twine R.. (2014). Temporal association of infant immunisation with pneumococcal conjugate vaccine on the ecology of *Streptococcus pneumoniae, Haemophilus influenzae* and *Staphylococcus aureus* nasopharyngeal colonisation in a rural South African community. Vaccine 32, 5520–5530. 10.1016/j.vaccine.2014.06.091 25101982

[B40] Park I. H. Kim K. H. Andrade A. L. Briles D. E. McDaniel L. S. Nahm M. H. (2012). Nontypeable pneumococci can be divided into multiple cps types, including one type expressing the novel gene pspK. MBio 3:e00035–12 10.1128/mBio.00035-12 22532557 PMC3340917

[B41] Pelton S. I. Huot H. Finkelstein J. A. Bishop C. J. Hsu K. K. Kellenberg J.. (2007). Emergence of 19A as virulent and multidrug resistant Pneumococcus in Massachusetts following universal immunization of infants with pneumococcal conjugate vaccine. Pediatr. Infect. Dis. J. 26, 468–472. 10.1097/INF.0b013e31803df9ca 17529860

[B42] Perez-Losada M. Alamri L. Crandall K. A. Freishtat R. J. (2017). Nasopharyngeal microbiome diversity changes over time in children with asthma. PLoS ONE 12:e0170543. 10.1371/journal.pone.0170543 28107528 PMC5249091

[B43] Pilishvili T. Lexau C. Farley M. M. Hadler J. Harrison L. H. Bennett N. M.. (2010). Sustained reductions in invasive pneumococcal disease in the era of conjugate vaccine. J. Infect. Dis. 201, 32–41. 10.1086/648593 19947881

[B44] Powers M. E. Wardenburg J. B. (2014). Igniting the Fire: *Staphylococcus Aureus* virulence factors in the pathogenesis of sepsis. PLoS Pathog. 10:e1003871. 10.1371/journal.ppat.1003871 24550724 PMC3923759

[B45] Quast C. Pruesse E. Yilmaz P. Gerken J. Schweer T. Yarza P.. (2013). The SILVA ribosomal RNA gene database project: improved data processing and web-based tools. Nucleic Acids Res. 41, D590–D596. 10.1093/nar/gks1219 23193283 PMC3531112

[B46] Reller L. B. Weinstein M. P. Werno A. M. Murdoch D. R. (2008). Laboratory diagnosis of invasive pneumococcal disease. Clin. Infect. Dis. 46, 926–932. 10.1086/528798 18260752

[B47] Rodenburg G. D. de Greeff S. C. Jansen A. de Melker H. E. Schouls L. M. Hak E.. (2010). Effects of pneumococcal conjugate vaccine 2 years after its introduction, the Netherlands. Emerg Infect Dis 16, 816–823. 10.3201/eid1605.091223 20409372 PMC2953990

[B48] Satzke C. Turner P. Virolainen-Julkunen A. Adrian P. V. Antonio M. Hare K. M.. (2013). Standard method for detecting upper respiratory carriage of *Streptococcus pneumoniae*: updated recommendations from the world health organization pneumococcal carriage working group. Vaccine 32, 165–179. 10.1016/j.vaccine.2013.08.062 24331112

[B49] Schloss P. D. Westcott S. L. Ryabin T. Hall J. R. Hartmann M. Hollister E. B.. (2009). Introducing mothur: open-source, platform-independent, community-supported software for describing and comparing microbial communities. Appl. Environ. Microbiol. 75, 7537–7541. 10.1128/AEM.01541-09 19801464 PMC2786419

[B50] Shahinas D. Tamber G. S. Arya G. Wong A. Lau R. Jamieson F.. (2011). Whole-genome sequence of *Streptococcus pseudopneumoniae* isolate IS7493. J. Bacteriol. 193, 6102–6103. 10.1128/JB.06075-11 21994930 PMC3194916

[B51] Shankar J. Nguyen M. H. Crespo M. M. Kwak E. J. Lucas S. K. McHugh K. J.. (2015). *Looking beyond respiratory* cultures: microbiome-cytokine signatures of bacterial pneumonia and tracheobronchitis in lung transplant recipients. Am. J. Transplant. 16, 1766–1778. 10.1111/ajt.13676 26693965

[B52] Sharma D. Baughman W. Holst A. Thomas S. Jackson D. da Gloria Carvalho M.. (2013). Pneumococcal carriage and invasive disease in children before introduction of the 13-valent conjugate vaccine: comparison with the era before 7-valent conjugate vaccine. Pediatr. Infect. Dis. J. 32, e45–53. 10.1097/INF.0b013e3182788fdd 23080290

[B53] Simoes A. S. Tavares D. A. Rolo D. Ardanuy C. Goossens H. Henriques-Normark B.. (2016). lytA-based identification methods can misidentify *Streptococcus pneumoniae*. Diagn. Microbiol. Infect. Dis. 85, 141–148. 10.1016/j.diagmicrobio.2016.03.018 27107535

[B54] Skov Sorensen U. B. Yao K. Yang Y. Tettelin H. Kilian M. (2016). Capsular polysaccharide expression in commensal streptococcus species: genetic and antigenic similarities to *Streptococcus Pneumoniae*. MBio 7:e01844–16. 10.1128/mBio.01844-16 27935839 PMC5111408

[B55] Tocheva A. S. Jefferies J. M. Rubery H. Bennett J. Afimeke G. Garland J.. (2011). Declining serotype coverage of new pneumococcal conjugate vaccines relating to the carriage of *Streptococcus pneumoniae* in young children. Vaccine 29, 4400–4404. 10.1016/j.vaccine.2011.04.004 21504773

[B56] Turner P. Hinds J. Turner C. Jankhot A. Gould K. Bentley S. D. (2011). Improved detection of nasopharyngeal cocolonization by multiple pneumococcal serotypes by use of latex agglutination or molecular serotyping by microarray. J. Clin. Microbiol. 49, 1784–1789. 10.1128/JCM.00157-11 21411589 PMC3122683

[B57] Turner P. Turner C. Jankhot A. Helen N. Lee S. J. Day N. P.. (2012). A longitudinal study of *streptococcus pneumoniae* carriage in a cohort of infants and their mothers on the Thailand-Myanmar border. PLoS ONE 7:e38271. 10.1371/journal.pone.0038271 22693610 PMC3365031

[B58] WHO and CDC (2011). Chapter 10: PCR for detection and characterization of bacterial meningitis pathogens: neisseria meningitidis, haemophilus influenzae, and *Streptococcus pneumoniae*, in Laboratory Methods for the Diagnosis of Meningitis Caused by Neisseria meningiditis, Streptococcus pneumoniae, and Haemophilus influenzae, 2nd Edn (Atlanta, GA: CDC and WHO Press), 105–156. Available online at: https://www.cdc.gov/meningitis/lab-manual/index.html

[B59] Weatherholtz R. Millar E. V. Moulton L. H. Reid R. Rudolph K. Santosham M.. (2010). Invasive pneumococcal disease a decade after pneumococcal conjugate vaccine use in an American Indian population at high risk for disease. Clin. Infect. Dis. 50, 1238–1246. 10.1086/651680 20367225

[B60] Zhou C. E. Smith J. Lam M. Zemla A. Dyer M. D. Slezak T. (2007). MvirDB–a microbial database of protein toxins, virulence factors and antibiotic resistance genes for bio-defence applications. Nucleic Acids Res. 35, D391–D394. 10.1093/nar/gkl791 17090593 PMC1669772

